# Nebulized fentanyl for respiratory symptoms in patients with COVID-19 (ventanyl trial)

**DOI:** 10.1097/MD.0000000000028637

**Published:** 2022-01-28

**Authors:** Nissar Shaikh, Mohamad Y. Khatib, Mohammad A. Al Wraidat, Ahmed S. Mohamed, Anood A. Al-Assaf, Abdul Gafoor M. Tharayil, Ahmad A. Abujaber, Abdulqadir J. Nashwan

**Affiliations:** Critical Care Medicine Department, Hazm Mebaireek General Hospital (HMGH), Hamad Medical Corporation (HMC), Doha, Qatar.

**Keywords:** cough, COVID-19, dyspnea, nebulized fentanyl, normal saline 0.9%, throat pain

## Abstract

Patients with coronavirus disease (COVID-19) commonly experience distressing and challenging respiratory symptoms. Interventions such as oxygen therapy, oral opiates, and traditional nebulizers like ipratropium bromide and salbutamol are variable in their efficacy, and therapy responses in patients are difficult to predict. The purpose of this study is to investigate the efficacy of nebulized fentanyl citrate on dyspnea, cough, and throat pain in patients with COVID-19 and evaluate the safety with any potential adverse events.

In COVID-19, about 59% of patients will exhibit cough, 35% generalized body ache and sore throat, and 31% dyspnea. Some methods such as nebulized lidocaine, magnesium sulfate, and systemic opioids have been used to manage the respiratory symptoms. It has been previously shown that fentanyl nebulizer has beneficial effect in improving shortness of breath in patients with chronic obstructive pulmonary disease. The proposed theory behind that was that fentanyl decreased the rate of spontaneous respiratory rate, diminished the brain stem chemoreceptor response to hypoxia and hypercarbia, in addition to exhibiting a modulating effect on the brain stem. Therefore, we hypothesize that nebulized fentanyl has superior effect in improving shortness of breath and relieving cough compared to normal saline, in addition to its advantageous throat pain relief, while exhibiting fewer side effects in patients with COVID 19 infection. Therefore, this phase-III, randomized, comparative, parallel assignment, single-blinded clinical trial aims at assessing the efficacy and safety of nebulized fentanyl to suppress cough, improve breathlessness, and relieve throat pain in patients with COVID-19.

## 1 Introduction

Respiratory symptoms of coronavirus disease (COVID-19) infection can be distressing to the patients and challenging to the physicians who are managing them. Studies have revealed that COVID 19 virus utilizes the angiotensin converting enzyme, a protein expressed on the surface of many cells including respiratory mucosal epithelium, as a port of entry.^[1–3]^ Angiotensin-converting enzyme-2 is normally secreted in upper and lower respiratory epithelial cells; thus, angiotensin-converting enzyme-2 stimulation in COVID-19 infection will significantly surge pro-inflammatory mediators and mucosal secretions, leading to dyspnea (breathlessness), cough, and throat pain.^[4,5]^

This protein also acts as a pro-inflammatory mediator, which contributes to increased respiratory secretions in respiratory infections, and some of the other symptoms, predominantly, dyspnea, cough, and throat pain.^[6,7]^ These symptoms variably respond to medical interventions such as oxygen therapy, oral opiates, ipratropium bromide and salbutamol nebulizers. Physicians commonly use antihistamines and antitussive medications to suppress cough. However, these cough medications cannot be delivered to the respiratory system in a nebulized form. The National Institute For Health And Care Excellence guidelines recommend using opioids in managing cough and shortness of breath in COVID 19 patients.^[8]^

Alternative medications to manage cough were nebulized lidocaine, magnesium sulfate, and systemic opioids.^[9]^ However, side effects of these medications make their use limited. For example, systemic opioids were associated with sedative effect, dizziness, nausea, vomiting, constipation, physical dependence, tolerance, and most seriously, their effect on depressing the respiratory drive.^[10]^ On the other hand, the nebulized opioids can presumably exhibit their desired effect while lacking many of the systemic side effects, by binding to the receptors (μ, κ, and δ) and some active peptides (enzymes necessary for activation of opioids) already present in the lung and small airways.^[11–14]^ Compared to morphine and dihydrocodeine, nebulized fentanyl is more potent with less depressing effect on the respiratory drive, and interestingly, with an equal potency to its intravenous form. Nebulized fentanyl as proven to relieve shortness of breath in patients with chronic obstructive pulmonary disease due to its effect on decreasing the rate of spontaneous respiratory rate, diminishing the brain stem chemoreceptor response to hypoxia and hypercarbia, and modulating the activity of the brain cortex.^[15]^

Thus, based on the above evidence, we hypothesize that nebulized fentanyl may suppress cough, respiratory drive, improve breathlessness or dyspnea with the added advantage of relieving throat pain with minimal adverse effects in COVID-19 patients. This is a novel study as there is no literature on using nebulized fentanyl in COVID-19 or other viral infections to suppress cough, relieve breathlessness or dyspnea, and relieve throat pain.

## 2 Objectives

Primary objectives: To assess the efficacy and safety of nebulized fentanyl to suppress cough, respiratory drive, improve breathlessness, and relieve throat pain in patients with COVID-19.

Secondary objectives: Overall patient outcomes (intensive care unit [ICU] or hospital stay and mortality).

Hypothesis: Nebulized fentanyl is effective and safe in providing symptomatic relief of cough, improving breathlessness, and relieving throat pain in patients with COVID-19, which is superior to saline nebulization.

## 3 Trial design

This is a phase-III, randomized, comparative, parallel assignment, single-blinded clinical study.

## 4 Methods: participants, interventions, and outcomes

### 4.1 Study setting

The study will take place in the medical ICU in Hazm Mebaireek General Hospital in Qatar.

### 4.2 Eligibility criteria

- Age: 18 to 65 years;
- Confirmed diagnosis (COVID-19) – positive polymerase chain reaction;
- Tachypnea (respiratory rate >30/min);
- Ability to provide informed consent and perform all study procedures.

### 4.3 Exclusion criteria

- History of allergy or adverse reaction to fentanyl or other opioids;
- Pregnancy;
- Active neuromuscular or musculoskeletal disease;
- Active malignancy;
- Morbid obesity (body mass index >40);
- Use of opioids in the previous 4 weeks;
- Inability to provide informed consent and perform all study procedures.

### 4.4 Outcomes measures

#### 4.4.1 Primary outcome measures

##### 4.4.1.1 Breathlessness

The severity of breathlessness will be measured by the 10-point Borg scale [time frame: 15-minutes posttreatment]. Patients will be instructed to use this scale to rate their breathing difficulty. The scale is ranged from 0 to 10, where 10 is the maximum breathing difficulty.^[16]^

##### 4.4.1.2 Throat pain

Throat pain will be graded by the Numerical Rating Scale, in which patients will be asked to circle the number between 0 and 10 that fits best to their pain intensity. Zero usually represents “no pain at all”, whereas the upper limit represents “the worst pain ever possible”.^[17]^

##### 4.4.1.3 Cough

The cough severity score represents a simple instrument, using a 10-point scale, where the patient can indicate the severity of their cough between the 2 extremes: 1 for no cough while 100 mm is the most severe cough.^[18]^

##### 4.4.1.4 Drug safety

Evaluate the safety of using the nebulized fentanyl with any potential adverse events.

#### 4.4.2 Secondary outcome measures

The overall patient outcomes (ICU or hospital stay and mortality).

### 4.5 Participant timeline

The trial is intended to last 1 calendar year following ethical clearance and will be extended yearly for a total of 5 years.

### 4.6 Sample size & recruitment

After randomizing the selected patients, a total of 200 patients will be recruited using the sequentially numbered, opaque, sealed envelope (SNOSE) technique: the randomization group will be written on paper and preserved in an opaque sealed envelope. A serial number will be printed on the envelope. Once the patient has agreed to participate, the investigator will open the sealed envelope and allocate the patient to the appropriate treatment group.

## 5 Methods: data collection, management, and analysis

### 5.1 Type and classification of study – randomized control trial

Baseline matching: Demographic variables and comorbidities will be compared between both groups. The severity of the disease will be compared using sequential organ failure assessment scores.

### 5.2 Intervention

Intervention group (n = 100): Administration of nebulized fentanyl (25 μg in 5 mL of normal saline) over 15 minutes, thrice daily using the traditional nebulizer. Treatment duration – 48 hours.

### 5.3 Control group

Administration of nebulized 0.9% saline solution (5 mL of normal saline), over 15 minutes, thrice daily using the traditional nebulizer. Treatment duration – 48 hours

Balancing measures: If nebulization does not provide symptomatic relief in these patients, oral antihistamines will be offered to inpatients, and dexmedetomidine will be considered for ICU patients. The assigned nurse will administer all medications.

### 5.4 Data management

The data gathered for this study will be uploaded to a secure database administered by the Hamad Medical Corporation IT staff (e.g., PACS). The sponsor will be the exclusive owner of the data. Source documentation will be made accessible to assist the electronic medical record. Clinical data will be recorded in each patient's source documents (i.e., the patient's medical record) by research employees. Adequate and accurate records will be kept in order to thoroughly document the research's conduct and to allow for later verification of the study results. After the research is completed, the investigators will keep all source documents, study-related papers, and data kept in the database dedicated to data collecting. As people are recruited in the experiment, data will be recorded.

### 5.5 Statistical methods

A well-structured case report form will be prepared and created to collect the essential data in line with the research study design and goals. Lead research investigators will conduct and maintain data quality in terms of completeness, verification, correctness, security, and confidentiality of data. Participants will be reassured that no personal identification will be used in scientific presentations or publications, that the anonymity and confidentiality of the study results will be maintained, and that their data will be kept confidential and accessible only to the study research investigators and authorized personnel from the research ethics committee.

The chi-square^[2]^ test and the Fisher exact test will be used to evaluate categorical data, if appropriate. The unpaired ‘*t*’ or Mann–Whitney *U* test will be used to compare continuous variables (quantitative data) between the 2 independent groups. The findings will be displayed together with the relevant 95% confidence interval. In addition, if necessary, other relevant regression models will be employed to examine and quantify the impact of various variables on primary and secondary outcome measures. Using appropriate statistical graphs, pictorial presentations of the relevant data will be created. All *P* values reported will be two-tailed, with *P* values less than .05 deemed statistically significant. All statistical analyses will be performed using the statistical software program SPSS 25 (SPSS Inc., Chicago, IL).

## 6 Methods: monitoring

### 6.1 Data monitoring & auditing

The clinical trial master file, as well as informed consent from patients, will be made available in accordance with the standards of the Joint Commission International, the Medical Research Center, and the Ministry of Public Health. Any side effects or adverse events that occur as a result of the experiment or are suspected to be associated to it will be reported to the hospital research committee and the Medical Research Center.

A registration report will be generated on a regular basis to track patient accrual and registration data completeness. A routine data quality report will also be prepared to evaluate missing data and discrepancies. The rate and amount of accrual, as well as the accuracy of evaluations and follow-up, will be evaluated on a regular basis during the research's duration, and possible concerns will be brought to the study team's attention for discussion and action. The study team may undertake random-template data quality and protocol compliance audits at least once per year, or more frequently if required. The Hamad Medical Corporation-Institutional Review Board, Ethics, and Data Safety Monitoring Board rules shall be followed for data safety and monitoring.

## 7 Ethics and dissemination

### 7.1 Research ethics approval

The study is submitted and under review by the Institutional Review Board-Medical Research Center at Hamad Medical Corporation in Qatar (MRC-01-21-798).

### 7.2 Protocol amendments

None.

### 7.3 Informed consent

The principal investigator will screen the patient pool, and those patients who meet the inclusion and exclusion criteria will be interviewed to discuss the experiment. The consenting procedure will begin with screening and then interviewing eligible patients to explain the study rationale, advantages, risks, and objectives, as well as their option to join or withdraw at any time without any consequences. The subject will be given time to select whether or not to participate in the study.

### 7.4 Confidentiality

To guarantee security, patients’ data will be coded and stored in a secure database with a unique username and password. The computerized charts and reports of the patients will be accessible only to the approved research team.

### 7.5 Access to data

Patients’ information will be moved to a secure database administered by the Hamad Medical Corporation IT staff (e.g., PACS). The sponsor will own any data generated as a result of this study.

### 7.6 Dissemination policy

The trial's findings will be published as mutually co-authored publications in peer-reviewed international medical journals and made freely available to the public.

## Author contributions

All authors have full access to the manuscript and take responsibility for the study design. All authors have approved the manuscript and agree with the submission.

**Conceptualization:** Nissar Shaikh, Mohamad Y. Khatib.

**Methodology:** Nissar Shaikh, Mohamad Y. Khatib, Mohammad A. Al Wraidat, Ahmed S. Mohamed, Anood A. Al-Assaf, Abdul Gafoor M. Tharayil, Ahmad A. Abujaber, Abdulqadir J. Nashwan.

**Project administration:** Abdulqadir J. Nashwan.

**Writing – original draft:** Nissar Shaikh, Mohamad Y. Khatib, Mohammad A. Al Wraidat, Ahmed S. Mohamed, Anood A. Al-Assaf, Abdul Gafoor M. Tharayil, Ahmad A. Abujaber, Abdulqadir J. Nashwan.

**Writing – review & editing:** Nissar Shaikh, Mohamad Y. Khatib, Mohammad A. Al Wraidat, Ahmed S. Mohamed, Anood A. Al-Assaf, Abdul Gafoor M. Tharayil, Ahmad A. Abujaber, Abdulqadir J. Nashwan.
